# Pragmatic Clinical Trial to Improve Screening and Treatment for Opioid Use Disorder in Primary Care

**DOI:** 10.1192/j.eurpsy.2023.1194

**Published:** 2023-07-19

**Authors:** R. Rossom

**Affiliations:** 1 HealthPartners Institute, Minneapolis; 2 University of Minnesota, Minneapolis

## Abstract

**Introduction:**

Opioid-related deaths continue to rise in the U.S. A clinical decision support (CDS) system to help primary care clinicians (PCCs) identify and treat patients with opioid use disorder (OUD) could help address this crisis.

**Objectives:**

To implement and test an OUD-CDS system in three health systems for the diagnosis and treatment of OUD in 90 primary care clinics.

**Methods:**

In this cluster-randomized trial, primary care clinics in three healthcare systems were randomized to receive or not receive access to an OUD-CDS system. The OUD-CDS system alerts PCCs and patients to elevated risk of OUD and supports OUD screening and treatment. It includes guidance on OUD screening and diagnosis, treatment selection, starting and maintaining patients on buprenorphine for waivered clinicians, and screening for common comorbid conditions. The primary study outcome is, of patients at high risk for OUD, the percentage receiving an OUD diagnosis within 30 days of index visit. Additional outcomes are, of patients at high risk for or with a diagnosis of OUD, (a) the percentage receiving a naloxone prescription, or (b) the percentage receiving a medication for OUD (MOUD) prescription or referral to specialty care within 30 days of an index visit, and (c) total days covered by a MOUD prescription within 90 days of an index visit.

**Results:**

The intervention started in April 2021 and continues through December 2023, with successful implementatio and uptake. PCCs and patients in 90 clinics are included; study results are expected in 2024.

**Image:**

**Figure:**
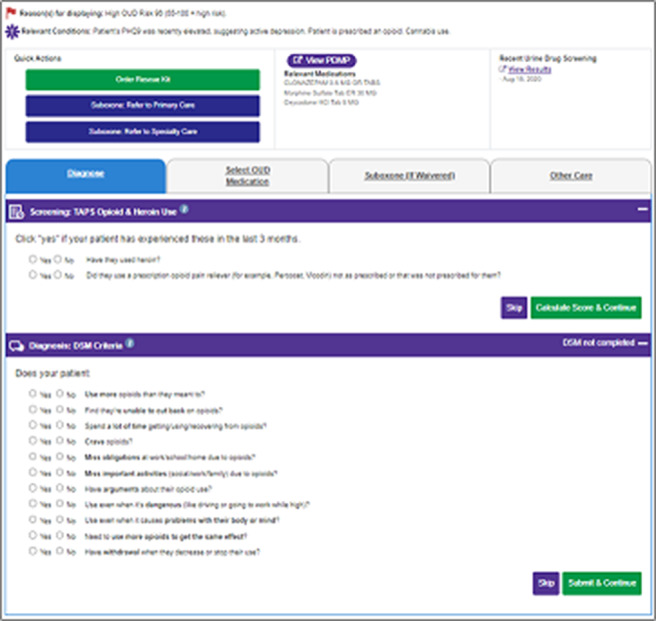

**Image 2:**

**Figure:**
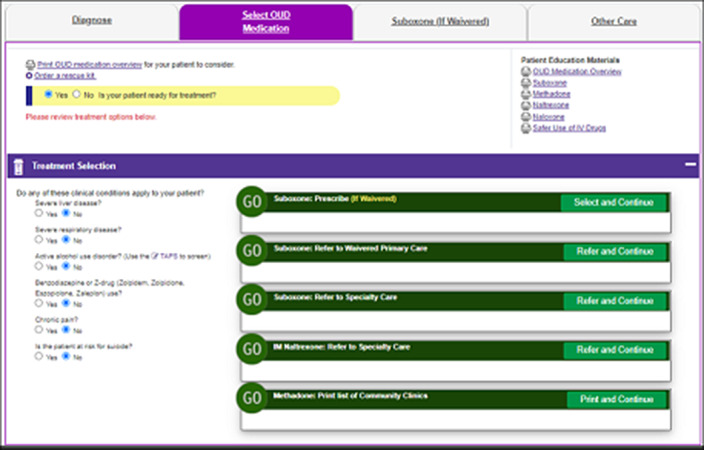

**Image 3:**

**Figure:**
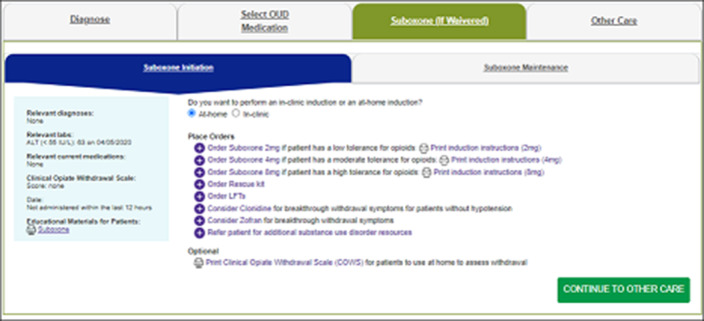

**Conclusions:**

If effective, this OUD-CDS intervention could improve screening of at-risk patients and rates of OUD treatment for people with OUD, a significant step in decreasing the morbidity and mortality associated with OUD.

**Disclosure of Interest:**

None Declared

